# Lived experience roles in forensic in-patient treatment

**DOI:** 10.1192/j.eurpsy.2021.87

**Published:** 2021-08-13

**Authors:** G. Drennan

**Affiliations:** South London And Maudsley Nhs Foundation Trust, Behavioural and Developmental Psychiatry Operational Directorate, Kent, United Kingdom

**Keywords:** Lived experience, co-production, Forensic, governance

## Abstract

wThe development of recovery-oriented practice in mental health has brought about a much greater prominence to the place of lived experience workers. Many aspects of indvidualised recovery-oriented care have been taken up in forensic mental health settings. However, the introduction of lived experience workers is arguably significantly more difficult when the dual vulnerabilities of forensic mental health services users are considered (Drennan & Alred, 2012). This paper will describe a multi-layered approach to the introduction of lived experience roles in a forensic in-patient unit. Roles have developed from being solely ward-based, to service-wide roles that include participation in management and service development, the creation of a Recovery College Forensic Campus, and to co-production and co-delivery of the psychological therapies programme. In addition to ‘mapping’ these developments in co-production, this paper will also describe the development of the governance structures that have been necessary to support this infrastructure. Lived experience workers require recruitment, vetting, placement, and aftercare, when they engage in the activities available. On-going mental health and risk stability cannot be assumed, and so regular formal and informal psychosocial support is required to ensure that workload pressures do not negatively impact on other service users and staff. The paper will suggest that much more attention needs to be paid to the development of organisational infrastructure to sustain and manage the growth of lived experience roles in forensic mental health settings than is currently in place.

**Disclosure:**

No significant relationships.

